# Predicting the Responses of Repetitively Firing Neurons to Current Noise

**DOI:** 10.1371/journal.pcbi.1003612

**Published:** 2014-05-08

**Authors:** Charles J. Wilson, David Barraza, Todd Troyer, Michael A. Farries

**Affiliations:** Department of Biology, University of Texas at San Antonio, San Antonio, Texas, United States of America; University of Pittsburgh, United States of America

## Abstract

We used phase resetting methods to predict firing patterns of rat subthalamic nucleus (STN) neurons when their rhythmic firing was densely perturbed by noise. We applied sequences of contiguous brief (0.5–2 ms) current pulses with amplitudes drawn from a Gaussian distribution (10–100 pA standard deviation) to autonomously firing STN neurons in slices. Current noise sequences increased the variability of spike times with little or no effect on the average firing rate. We measured the infinitesimal phase resetting curve (PRC) for each neuron using a noise-based method. A phase model consisting of only a firing rate and PRC was very accurate at predicting spike timing, accounting for more than 80% of spike time variance and reliably reproducing the spike-to-spike pattern of irregular firing. An approximation for the evolution of phase was used to predict the effect of firing rate and noise parameters on spike timing variability. It quantitatively predicted changes in variability of interspike intervals with variation in noise amplitude, pulse duration and firing rate over the normal range of STN spontaneous rates. When constant current was used to drive the cells to higher rates, the PRC was altered in size and shape and accurate predictions of the effects of noise relied on incorporating these changes into the prediction. Application of rate-neutral changes in conductance showed that changes in PRC shape arise from conductance changes known to accompany rate increases in STN neurons, rather than the rate increases themselves. Our results show that firing patterns of densely perturbed oscillators cannot readily be distinguished from those of neurons randomly excited to fire from the rest state. The spike timing of repetitively firing neurons may be quantitatively predicted from the input and their PRCs, even when they are so densely perturbed that they no longer fire rhythmically.

## Author Summary

Most neurons receive thousands of synaptic inputs per second. Each of these may be individually weak but collectively they shape the temporal pattern of firing by the postsynaptic neuron. If the postsynaptic neuron fires repetitively, its synaptic inputs need not directly trigger action potentials, but may instead control the timing of action potentials that would occur anyway. The phase resetting curve encapsulates the influence of an input on the timing of the next action potential, depending on its time of arrival. We measured the phase resetting curves of neurons in the subthalamic nucleus and used them to accurately predict the timing of action potentials in a phase model subjected to complex input patterns. A simple approximation to the phase model accurately predicted the changes in firing pattern evoked by dense patterns of noise pulses varying in amplitude and pulse duration, and by changes in firing rate. We also showed that the phase resetting curve changes systematically with changes in total neuron conductance, and doing so predicts corresponding changes in firing pattern. Our results indicate that the phase model may accurately represent the temporal integration of complex patterns of input to repetitively firing neurons.

## Introduction

Some neurons fire repetitively in the absence of any input, and many others show repetitive firing with sufficient tonic excitation. Because the same neuron may be driven to fire by a large transient synaptic input either from the rest state or when firing repetitively, often no strong distinction is made between the two. However, a neuron responds to subthreshold inputs in fundamentally different ways, depending on whether it is at rest or firing repetitively. Inputs to a repetitively firing neuron need not insert or delete spikes from the ongoing pattern, but instead may alter the timing of spikes that would have occurred anyway. In doing so, an input may disturb a rhythmic pattern of firing and replace it with a less regular pattern at about the same rate.

Temporal integration of subthreshold inputs in repetitively firing neurons differs in several ways from that seen in cells driven to fire from rest. The window of temporal summation in repetitively firing neurons is not constrained by the membrane time constant; inputs arriving at any time during an interspike interval may influence the timing of the next spike [1]. The effectiveness of inputs in altering spike timing depends not only on their sign and size, but also on their time of arrival during the interspike interval, as represented in the cell's phase resetting curve (PRC). The PRC is usually measured by applying an isolated subthreshold synaptic input or current pulse at various times after a spontaneous action potential and observing its average effect on the timing of the next action potential [1]. Measurement of phase resetting has been performed in a number of different cell types, which show a range of different sensitivity profiles during the interspike interval [2], [3], [4], [5], [6]. These differences reveal a spectrum of cell-type specific strategies for temporal integration among repetitively firing cell types in various parts of the brain. The sensitivity of repetitively firing neurons to specific patterns of inputs in time, their phase-locking to periodic inputs, and synchronization of coupled networks of repetitively firing neurons are all determined by details of phase resetting curve shape [7].

Neurons whose responses to inputs can be represented by their PRCs may be amenable to representation by a phase model. In this simple neuron model, the cell's unperturbed rhythmic firing is conceptually a closed path in a multidimensional space whose dimensions are the membrane potential and activation and inactivation states of all of the participating ion channels [e.g. 8]. This state space may be very complex, but the trajectory followed by a repetitively firing neuron is much simpler. It can be reduced to a circle, and the cell's state described by a single variable, phase, which is the angular location of the cell on its closed trajectory. The cell is assumed not to stray far off of this path in response to low amplitude stimuli, so their effect may be approximated by shifts in the location of the cell along its trajectory – changes in phase. For the phase model simplification of the neuron, the PRC and average firing rate comprise a complete description of the response to subthreshold transient inputs.

Application of the phase model to real neurons is impeded by the fact that phase is not an experimentally measurable variable. The phase of a neuron is manifest only at the time of action potential generation; at all other times it is inferred, and so is called “latent phase” [9]. During rhythmic firing in the absence of any phase-resetting input, time and phase evolve together, and so time can be used as a surrogate measure of phase. The usual method for constructing the phase-resetting curve takes advantage of this by applying only one phase-altering input per firing cycle, so the phase of the cell at the time of the stimulus may be estimated from its timing. But after any phase-altering stimulus, time and phase become dissociated, and the phase at which a second stimulus is presented cannot be directly estimated from its time of delivery. For complex sequences of excitatory and inhibitory inputs, as often occurs in neurons in situ, the dissociation of phase and time becomes severe. Thus, the use of time as a surrogate for phase fails in direct proportion with the effectiveness of the input pattern in sculpting spike timing. Validating the phase-resetting approach in real cells requires a demonstration that the PRC can be used to predict the responses of neurons to temporally complex stimuli that disrupt phase substantially during individual interspike intervals.

We have tested the applicability of the phase model to predict spike times in subthalamic nucleus (STN) neurons recorded in tissue slices. The STN neuron is an autonomously firing cell in the basal ganglia whose phase-resetting curve has been previously characterized [10] and derived from its biophysical properties [11]. We used intracellular injection of a series of contiguous brief pulses whose amplitudes were each independently drawn from a Gaussian distribution with zero mean. Using a symmetrical noise stimulus enables a different strategy for measuring the effect of the stimulus on spike timing. The mean change in spike time caused by a symmetrical noise stimulus is expected to be zero. Instead, the effect of the stimulus is seen as a change in the variability of spike timing.

We used a phase model to predict interspike intervals of STN neurons during stimulation, and adapted the approximation of Ermentrout et al. [12] to predict the coefficient of variation of interspike intervals in subthalamic neurons as a function of the amplitude and duration of the noise pulses and neuronal firing rate. Our results indicate that the phase-based simplification of the STN neuron, and perhaps other repetitively firing cells, can accurately predict responses to temporally complex trains of inputs even when the perturbations in timing are large enough to obscure the oscillatory nature of the neuron's firing. They also suggest that the unconventional properties of temporal integration associated with this model may govern their ongoing firing patterns in the intact basal ganglia circuit.

## Results

### The Phase Model

We employed a simple model of a repetitively firing neuron, in which firing is periodic in the absence of input. Within each cycle, the trajectory of the membrane potential and the states of all the voltage-dependent channels participating in repetitive firing are projected into a single dimension, phase [9]. In the absence of stimuli, phase increases linearly in time, starting from a value of zero and ending at a value of 1 over the course of the interspike interval. In a conductance-based model of a neuron, phase may be measurable as the angle along a closed multidimensional trajectory in the neuron's phase space, scaled so that equal angles subtend equal amounts of time [8]. The phase model gains simplicity by assuming that the neuron's path never deviates too far from its periodic trajectory in that space, so that stimulus-generated perturbations can be treated as transient changes in the rate of advance in phase. When a stimulus terminates, the cell continues on its original trajectory, but retaining any net advance or delay in phase accumulated during the stimulus. All the complexity of the neuron's interspike trajectory, changes in the amplitude and sign of voltage-sensitive conductances and regions of slow and fast evolution of the membrane potential, are encapsulated in the infinitesimal phase resetting curve (PRC). The PRC gives the sign and sensitivity of the neuron's phase-rate response to perturbing currents. Phase evolves as
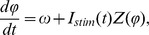
in which ϕ is phase, ω is the constant rate of drift in phase measured as the firing rate of the neuron in the absence of any perturbation, I_stim_(t) is the time-varying current applied to the neuron, and Z(ϕ) is the phase-dependent responsiveness of the cell – its infinitesimal phase resetting curve. Positive and negative stimulus currents produce equal-sized but opposite-sign effects on rate of change of phase. This is a simple model, but some of the simplicity is illusory, because time and phase are both variables and, while related, become uncoupled whenever there is a stimulus current. Stimulus current is defined in time, but the PRC is a function of phase.

### Predicting Spike Time Variability

The effect of noise can be measured by its effect on the variability of firing, measured as the coefficient of variation of intervals. Ermentrout et al. [12] derived an approximation for the evolution of the variance of the latent phase distribution for a phase model subjected to Gaussian white noise. In their approximation, the variance of the phase distribution at the time of the average interspike interval is equal to the variance of the Gaussian white noise times the integral of the square of the PRC. Their treatment shows that for small amplitude noise the variance of the interspike interval distribution is equal to the variance of the phase distribution at the time of the average interval, scaled by 1/ω^2^. The scaling by frequency occurs because the conversion from average phase to average time is accomplished by multiplying by the period, which is the reciprocal of frequency. Because the spike times are scaled by 1/ω, the variance of spike times is scaled by 1/ω^2^.

Before applying the model to the analysis of experimental data, we used a Monte Carlo simulation to examine the ability of the Ermentrout et al. analysis, which is based on theoretical white noise, to predict interspike interval variability caused by finite current pulses like the ones used in our experiments. We simulated a repetitively firing neuron using a discrete-time simulation of the phase model, with a time step of 1 ms and firing rate varying from 0.25 to 5 Hz. The current noise consisted of a series of contiguous current pulses of fixed duration, like those in our neurophysiological experiments. The PRC used for these simulations was derived from a simple neuron model [13], which is qualitatively similar to that of STN neurons (see below). The amplitude of each current pulse was independently drawn from a Gaussian distribution with zero mean. The duration and standard deviation of the distribution for the noise pulses remained fixed during any simulation, but was varied across simulations. We varied the duration of the pulses from 1 to 10 ms and the standard deviation of the noise from 25 to 800 pA. In each simulation, 5000 trajectories were collected, so that a frequency distribution of phase could be constructed at each time step.

**Figure 1:**
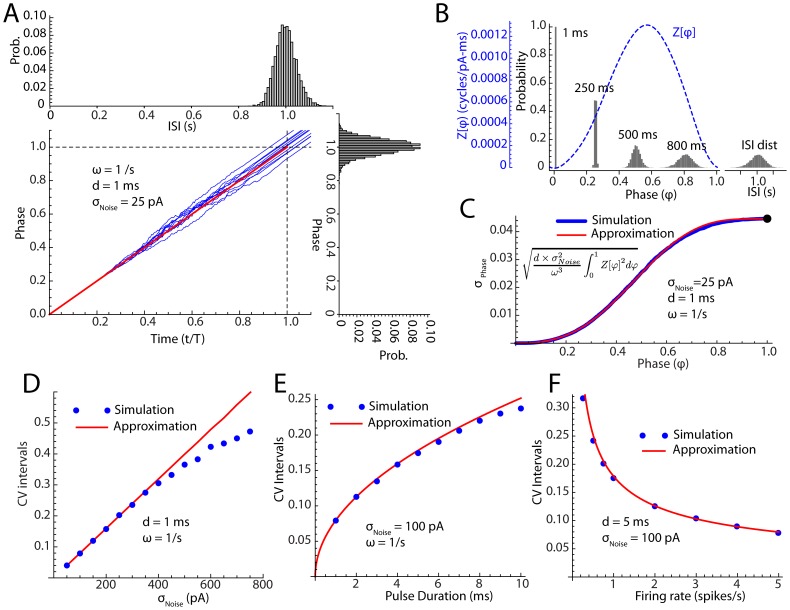
The effect of noise on the variability of spike times in the phase neuron model. A. The relationship between phase trajectories, phase distribution at the mean spike time, and the distribution of interspike intervals. Ten example phase trajectories are shown in blue. The red line is the mean of 5000 trajectories. The distribution of phases at the firing time (average phase = 1) is shown in the histogram to the right of the trajectories and the histogram of interspike intervals is shown above. B. The evolution of the phase distribution for 5000 trajectories like the ones in A. The mean moves at the drift rate ω but the variance increases depending on the phase resetting curve Z (shown in blue). The variance of the interspike interval distribution is the same as that of the phase distribution at the firing time, scaled by 1/ω^2^. C. The evolution of the standard deviation of the phase distribution (blue line) for the Monte Carlo simulation of the phase neuron, and the Ermentrout et al. approximation to that evolution (red line). The large black dot is the standard deviation of the interspike intervals (for mean firing rate of 1 spike/s). The approximation for standard deviation of phases is shown in the inset. D–F Effect of noise amplitude, pulse duration, and baseline firing rate on the coefficient of variation of interspike intervals in the Monte Carlo simulation (blue points) compared to the prediction of the approximation (red line).

The evolution of phase in a typical simulation is shown in Figure 1A (lower panel). Ten phase trajectories are shown with the mean phase trajectory superimposed. Individual phase trajectories did not deviate far from the mean trajectory in the early part of the interspike interval, because the effect of noise was limited by the low value of the PRC during that part of the trajectory (Figure 1B). As the PRC grew, the phase trajectories became more sensitive to the noise and deviated from each other and from their average. In the late part of the interspike interval a decrease in the value of the PRC caused the trajectories to maintain their relative positions and to progress in parallel toward the firing point with slope ω. The times at which each neuron crossed the firing point (phase = 1) were used to construct an interspike interval histogram (Figure 1A, top panel). In these simulations (unlike real neurons), neurons were not re-injected to generate a new phase trajectory at the end of the interspike interval, but were allowed to continue under the influence of the constant drift rate ω. This sets up an exact mapping between phase values greater than 1 and the time since the spike. Vertical slices through the phase trajectories in Figure 1A were used to construct distributions of latent phase at various times in the interspike interval (Figure 1B).

We used current pulses of durations comparable (when compared to the period of firing) to those that could be practically delivered in our neurophysiological experiments. To compare the Ermentrout et al. approximation to our simulations, it was necessary to modify their approximation to account for the finite bandwidth of the pulse noise used in our simulation. For a set of k noise pulses of duration d, the amplitude of each of which is drawn from a Gaussian distribution with variance σ_Noise_^2^, the effective noise variance is k*d^2^* σ_Noise_^2^. For contiguous pulses, the number of pulses (k) is the mean interspike interval divided by pulse length, (1/d ω). Therefore the rate of charge accumulation, measured in units of charge^2^ per interspike interval, is d* σ_Noise_^2^/ω. The coefficient of variation of interspike intervals (CV) predicted by the Ermentrout et al. [12] approximation for our simulations, (and our experimental results) is:
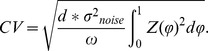



The Ermentrout et al. approximation assumes that the probability distribution of phase stays narrow enough so that the value of the PRC at the mean of the distribution for any time point can be used as a good estimate of its value everywhere in that distribution. Examination of Figure 1B shows that this assumption is not strictly met, even for the low noise level used in that example, but a comparison of the approximation with the measured evolution of the standard deviation of phase shows very good agreement anyway (Figure 1C, red and blue lines). Because of the relationship between interspike interval variance and the variance of the phase distribution at t = 1/ω, the approximation also continues to be accurate for the coefficient of variation of intervals (Figure 1C, large black dot at phase = 1).

We compared our Monte Carlo simulations with the Ermentrout et al. approximation for a wide range of noise levels, pulse durations, and firing rates. The approximation predicts a linear relationship between the CV of intervals and the standard deviation of the applied noise pulses. At high noise levels, the approximation is expected to fail, because the phase distribution becomes broad (especially at late phases) and all trajectories cannot be treated as being at the same point in phase for purposes of calculating the effect of a pulse of noise applied at any particular time. The range over which the assumption holds is also expected to be dependent on the shape of the phase resetting curve. For the phase resetting curve used in these simulations, the linear relationship between CV of intervals and σ_Noise_ held over a wide range of noise levels, predicting CVs accurately up to about 0.4 (Figure 1D). For values of noise amplitude at which the approximation was good, varying the pulse duration d produced the square root relationship predicted by the approximation (Figure 1E), and changing the firing rate produced the predicted reciprocal square root relationship (Figure 1F).

### The Effect of Symmetric Current Noise on STN Neuron Firing

We tested the effect of Gaussian noise pulses of on 89 STN neurons recorded in the perforated patch configuration. Perforated patch recording was required to maintain stable autonomous firing at constant rate over the course of the experiment, which lasted at least 30 minutes, and was typically more than an hour. In 21 cells we applied Gaussian pulsed noise in several different combinations of pulse duration and amplitude, consisting of contiguous pulses varying from 0.25 ms to 2 ms duration, and from 10 to 100 pA in standard deviation. In 12 additional cells, the noise was fixed at a standard deviation of 60 pA and 0.5 ms duration, and firing rate was altered by passage of constant current while applying noise. In 9 other cells, we applied an artificial leak conductance during application of current noise. In 47 additional cells, we compared PRCs obtained using current noise versus synaptic stimulation.

**Figure 2:**
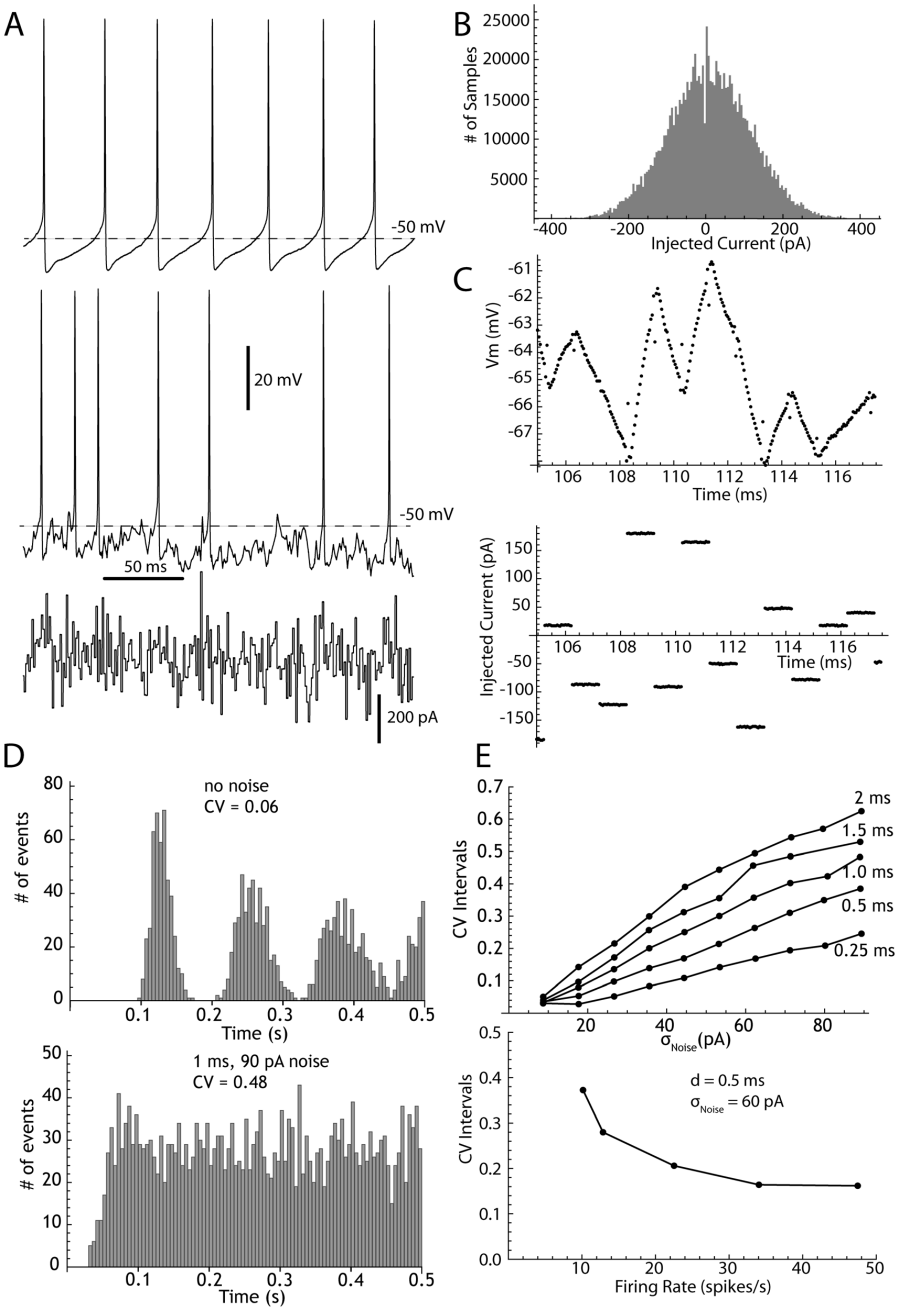
Responses of STN neurons to pulsed current noise. A. Autonomous firing of a STN neuron recorded in the absence of injected noise (upper panel) and in the presence of contiguous 1 ms current pulses (middle panel). The current injected is shown in the bottom panel. The standard deviation of the noise in this example was 90 pA. Average firing rate was unchanged. B. Histogram showing the distribution of noise pulse amplitudes. C. Higher resolution of the injected current (lower panel) and the membrane potential response to currents (upper panel) in the same cell. The capacitative transient at pulse onset and offset is restricted to a single sample (0.05 ms), the membrane changes are almost entirely capacitative and series resistance is well-compensated. D. Changes in regularity of firing during application of the same noise shown in A–C, as indicated by the autocorrelation histogram in the absence (upper panel) and the presence (lower panel) of noise. E. An example showing dependence of interspike interval CV qualitatively similar to those predicted from the phase model, for noise amplitude, pulse duration and baseline firing rate.

For the 21 cells used to study the effects of noise pulse duration and amplitude, each noise episode lasted 1 minute. As previously reported (for review see 14), STN neurons fired continuously in the absence of any applied current, with rates ranging from 4.5 to 31.9/s (mean = 12.07, sd = 6.3, n = 89), and had coefficients of variation in the absence of injected current ranging from 0.04 to 0.35 (mean = 0.11, sd = 0.06, n = 89). An example is shown in Figure 2 (A–E). Gaussian current pulses were generated in real time using a pseudo-random noise generator. Both the injected current and the membrane potential were digitized and recorded during data acquisition (Figure 2A and B). Application of current pulses (Figure 2A bottom) produced voltage perturbations in the membrane potential of the neurons and profoundly disrupted the pattern of their repetitive firing (Figure 2A middle trace). It was critical for our quantitative comparisons that the stimulus current pulses actually delivered their charge to the neuronal membrane, and none was lost through the stray capacitance of the amplifier or micropipette. The use of pulses in the 0.5–2 ms range allowed us to monitor and ensure the charge was delivered as commanded, despite the limited bandwidth of our recording amplifier. Electrode capacitance and series resistance compensation were monitored and corrected continuously throughout the experiment, using the experimental noise pulses. A typical response of a STN neuron to current pulses is shown in Figure 2C. Beyond ensuring that charge was not lost charging stray capacitances, we were concerned that some might be lost by dissipation through the membrane resistance. To minimize this, we used current pulses brief enough so that the voltage transients remained approximately linear, as shown in Figure 2C, top trace. We measured these slopes to ensure that each current pulse produced a transient whose slope was proportional to the amplitude of the current pulse.

Gaussian pulsed current noise in the range used in our experiments produced profound changes in the firing pattern of STN neurons. An example is shown in Figure 2. In the absence of any stimulus, the autonomous firing of STN neurons was very regular, as evident in the example shown in Figure 2A, the periodic autocorrelation in Figure 2D (top) and a CV of about 0.04. Injection of 1 ms pulses drawn from a Gaussian distribution with standard deviation of 90 pA produced the aperiodic firing pattern indicated by the autocorrelation shown in Figure 2D (bottom) in the same neuron (and a CV of about 0.45), with less than 10% change in firing rate. Noise increased the irregularity of firing measured by CV in all STN neurons tested, and CV increased approximately linearly with the standard deviation of the noise, as shown for an example in Figure 2E (top). Increases in the pulse duration produced changes in the slope of the relationship between CV and σ_Noise_. The change in this relationship was sub-linear with duration, consistent with the square root relationship predicted by the approximation.

In an additional set of experiments we modified the firing rates of 12 neurons over a range from about 5 to 50 spikes/s using long duration application of constant current [14] and there was a strong inverse relationship between CV and firing rate, resembling that in the Monte Carlo experiments, as shown in the example in Figure 2E (bottom).

Although these results were qualitatively consistent with the predictions of the phase model, they were not sufficient for a quantitative comparison with the model's predictions. The slope of the CV/σ_Noise_ relationship was very constant within cells, but varied widely from one neuron to the next in a way that could not be accounted for by current pulse duration or firing rate. This indicated that the cells varied in their sensitivity to noise, meaning that they had different PRCs. This was consistent with our previous observation that STN neurons, although very similar in their resting firing patterns, vary widely in both shape and overall size of their PRC (10). We concluded that we could not use a single average PRC to reconstruct the responses of all STN neurons. It was necessary to measure the PRC of each neuron and estimate the Sensitivity (integral of the square of the PRC, as in the Ermentrout et al. approximation) on a cell-by-cell basis. Because we had injected current noise into these neurons, we adapted a method of measuring the phase resetting curve from the response to injected noise.

### Phase Resetting Curves of STN Neurons Measured Using Gaussian Noise

There are a number of different methods for measuring a cell's PRC, including several that are based on injection of current noise [e.g.15]. We modified a multiple regression method outlined by Netoff et al. [16] (see Methods). In our version of this method, each interspike interval is divided into a set of discrete equal-sized phase bins, and the charge delivered within each is treated as an independent predictor variable regressed against the normalized length of the corresponding interspike interval. Each of these yields a slope for the Δphase/charge relationship that is a point on the PRC. The standard errors for the slopes obtained from the regression allow a direct measure of reliability of each point on the PRC, and the proportion of the variance in ISI that can be accounted for in the regression is obtained as R^2^. It should be noted that the standard errors obtained by this method are not a measure of the variance of the PRC, as described in Ermentrout et al. [12], and they do not vary in the same way with the amplitude or slope of the phase resetting curve. They are standard errors of the estimates for the PRC obtained in the regression.

We used the method described above to measure the PRC, initially for our sample of 21 neurons studied with a broad range of noise amplitudes and pulse durations. We constructed the PRC for each 60 s. sample taken at each noise amplitude and duration. We restricted our analysis to current pulses of 1 ms or less, to increase the phase-resolution of our results.

**Figure 3:**
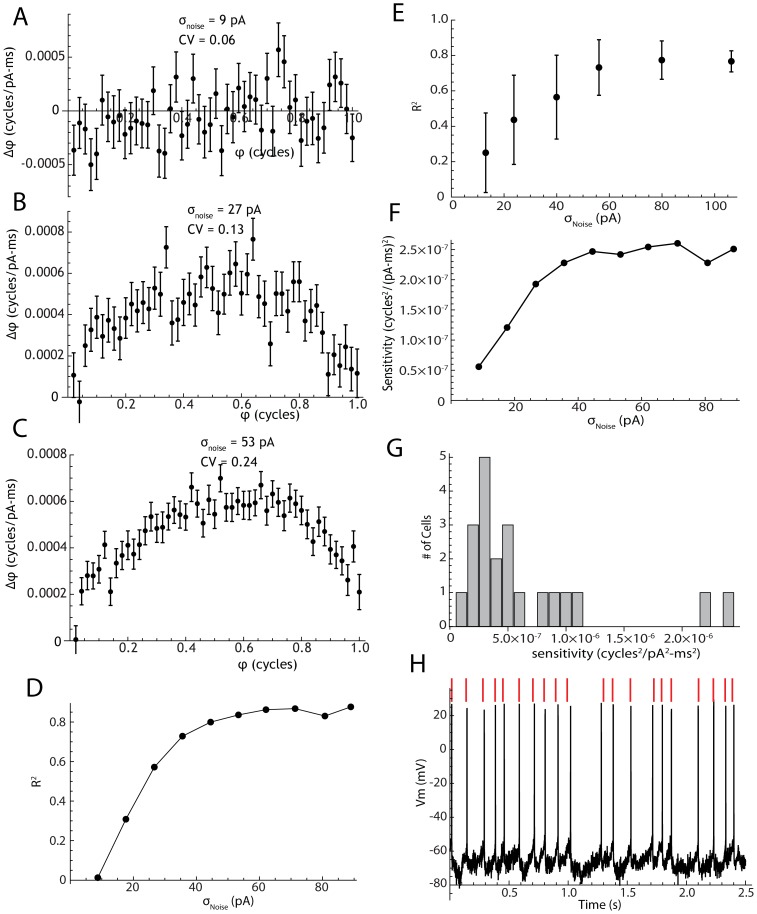
Predicting spike times from the PRC. A–C. Examples of phase resetting curves calculated in a single neuron for σ_Noise_ from 9 to 50 pA. The estimate of the PRC improves as σ_Noise_ is increased, up to about 50 pA, and then is stable. D. The proportion of ISI variance accounted for by the injected noise (R^2^ for the regression) increases and then stabilizes near 80%. E. For the group of 21 neurons tested, the proportion of the ISI variance accounted for by the noise follows a similar profile. Noise levels of 60 pA and above were equivalent, with ≈80% of the ISI variance being predicted by the phase model interacting with the injected noise. F. The measurement of Sensitivity (integral of the square of the PRC) increases in parallel with R^2^, and is constant at higher levels of injected noise. G. Histogram of Sensitivities for neurons in the sample. H. The phase model predicts spike times with high accuracy. A sample of intracellular recording is shown, with action potentials times predicted by the phase model shown by dashed red lines.

STN neurons do not fire perfectly regularly, even in the absence of injected noise. To measure a PRC, a substantial proportion of the variance in interspike intervals must be accountable to the injected noise. We used R^2^ for the regression to estimate the degree to which the variability in interspike interval was attributable to the injected current noise. A typical series of phase resetting estimates obtained at different levels of injected noise is shown for one STN neuron in Figure 3(A–C). At the lowest noise levels it was evident that the noise did not perturb the firing significantly (Figure 3A, 9 pA), whereas higher noise levels produced phase resetting curve estimates with similar shapes (Figure 3B–C, 27 & 53 pA) but decreasing standard errors. The proportion of interspike interval variance that could be accounted for by multiple linear regression with the injected noise (R^2^) rose rapidly between 9 and 40 pA, and increased only slightly beyond that point (Figure 3D). This increase and subsequent saturation in the proportion of variance accounted for by the noise was consistent across the sample of 21 cells for which we varied the noise amplitude (Figure 3E). Rarely did the injected noise ever achieve R^2^ values beyond about 0.85. Most of the remaining variance was no doubt due to intrinsic noise that is responsible for the resting variability in firing. Other variance could arise from factors not accounted for by the phase model of the neuron, i.e. interactions between noise pulses or interactions between ISIs.

We used the individualized phase resetting curves for each neuron to calculate its Sensitivity (the integral of the square of the phase resetting curve). The estimate of Sensitivity increased in parallel with the R^2^ at low currents, and was stable over a range of noise amplitudes from about 40 pA to 100 pA (Figure 3F). The distribution of Sensitivities for the neurons in this sample is shown in Figure 3G. Most neurons fell between 1×10^−7^ and 1×10^−6^ cycles^2^/(pA^2^-ms^2^), although there were two neurons with higher Sensitivities. Although these were outliers, we could find no evidence that there was anything wrong in their measurement, and all cells in the sample were retained for further analysis.

### Influence of PRC Shape on Spike Timing

Having calculated the PRC, simulations of the phase model could be used to predict the time between spikes for any pattern of pulsed current noise. An illustration of such a prediction is shown in Figure 3H. For this figure, a PRC was obtained using the linear regression method. Using another data series from the same cell, we calculated the sequence of injected noise pulses as input to a phase model based on this PRC. Restarting the phase model for each action potential, we produced a predicted phase evolution which, when it attained a value of 1, predicted the time of the subsequent spike. The red lines indicate the resulting predicted interspike intervals, superimposed over the recorded voltage trace. For each of the 21 cells in our sample, we used the PRC used for calculation of Sensitivity to predict the interspike intervals in a separate data episode from the same cell (the one with the next higher noise amplitude in the series). These predictions were highly accurate, with the prediction accounting for 68%–87% of the variance in interspike intervals (mean = 74.6%, sd = 8.6%). To determine how cell-to-cell variations in PRC shape would affect the accuracy of these spike time predictions, we compared the predictions calculated from each cells PRC with spike times predicted using the generic type 1 PRC used in our Monte Carlo simulations. To make the comparison based on variations in PRC shape, and not size, we scaled the generic PRC so that it had the correct Sensitivity for each cell. Even though the generic PRC had approximately the same overall shape as the PRCs of most STN neurons, it was significantly less accurate at predicting spike timing (t = 7.6, df = 20, p<0.01).

### Comparison with Phase Resetting Curves Derived from Synaptic Stimulation

**Figure 4:**
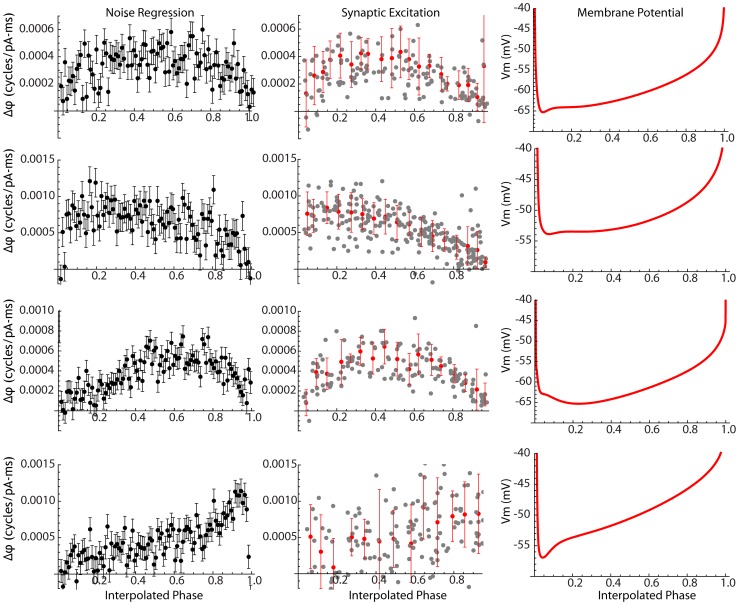
The range of PRC shapes seen in STN neurons. Phase resetting curves calculated by noise regression are in the left column. The phase resetting curve obtained using synaptic stimulation is shown in the center column, and the phase-normalized average interspike membrane potential trajectory is shown in the right hand column. Phase resetting curves measured in the two ways are similar, and are associated with differences in the average membrane potential trajectories.

**Figure 5:**
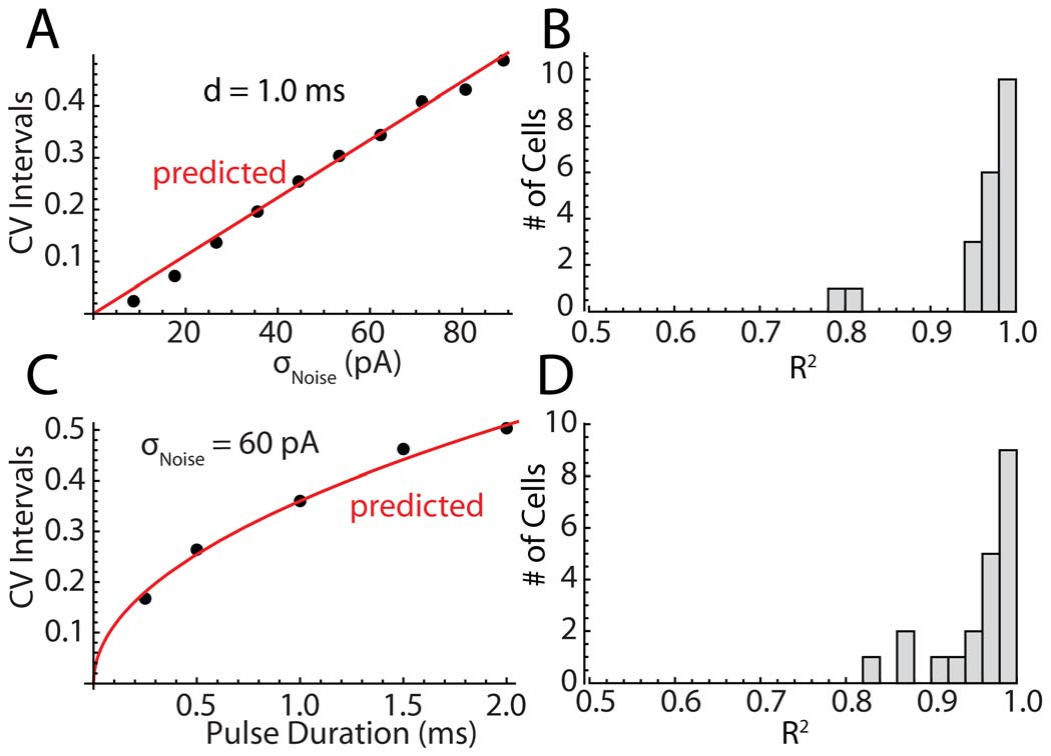
Prediction of CV in STN neurons with changes in noise amplitude and duration. A. An example showing the relationship between CV and noise amplitude for 1's sensitivity is in red. Goodness of fit was measured as the proportion of the variance in the data accounted for by the approximation. B. Distribution of goodness of fit for the CV versus noise amplitude for the sample of 21 neurons. C. The relationship between CV and pulse duration for the same cell as in A, displayed with the prediction. D. The distribution of goodness of fit for pulse duration across neurons in the sample.

We have previously reported that the PRC measured with isolated current pulses may be an imperfect predictor of the phase resetting effect of synaptic inputs [10]. To test the phase resetting curve obtained using our noise regression method against those measured using synaptic stimulation, we measured PRCs from 47 cells using both methods. For synaptic stimulation, we electrically stimulated the internal capsule rostral to the STN in the presence of GABA_A_ and GABA_B_ antagonists (as in [10]). The synaptic phase resetting curves, measured as phase changes per voltage change in membrane potential were normalized to charge for comparison to the noise-generated curves, using the effective cell capacitance seen measured from synaptic potentials as described by Farries and Wilson [10]. Examples showing phase resetting curves measured using both methods are shown in Figure 4. As previously reported [10], STN neurons do not all have the same phase resetting curve, although all of them are of Type 1, lacking a measurable negative lobe. Variation in phase resetting curves among neurons includes both changes in overall amplitude (e.g. compare Figure 4A &B) and changes in the shape of the curves. These difference in phase resetting curves are were not attributable to sampling error or a bias in the method of measurement, being consistent when measured using both methods. They are also reflected in the shape of the phase-interpolated membrane potential trajectory during the interspike interval (shown at right for each example cell in Figure 4). The decay of the phase resetting curve toward zero at late phases is determined by the steepness of the membrane potential trajectory [11]. The phase resetting curves of cells whose voltage trajectories are highly scoop-shaped, and become steep early show a more gradual decay to zero. In contrast, cells whose voltage trajectories are ramp-like, or concave-down have shallow slopes over much of the trajectory, and a sudden increase in slope at the onset of an action potential. The latter have lower-valued PRCs at early phases, rising toward the end and having a sudden crash to zero at the time of spiking (compare Figure 5B and D). As reported previously [10], this variation in PRC shape was not correlated with variation in spontaneous firing rate. Although STN neurons share a common set of ionic conductances responsible for their autonomous firing (for review see [14]), they apparently differ enough in relative strengths to produce a range of subtly different interspike membrane potential trajectories and PRCs.

Quantitative comparison of the exact shapes of PRCs obtained using the two methods is troubled by the high variance of the synaptic PRC. We therefore calculated the Sensitivity, the integral of the square of the phase resetting curve, and also the centroid (first moment) of the PRCs in each neuron measured using each method. Sensitivities and centroids measured with the two methods were highly correlated (r = 0.77, p<0.01 and r = 0.51, p<0.01).

### Predicting CV from Phase Resetting Curves

Having established a method for calculating the PRC and Sensitivity for neurons from the ongoing response to noisy current pulses, we tested the ability of the Ermentrout et al. approximation to predict how noise amplitude, pulse duration, and baseline firing rate affect the response variability of the neuron as measured by the CV.

For noise amplitude and pulse duration, we analyzed the responses of 21 neurons whose responses we had collected for varying levels of noise amplitude and pulse duration. For each neuron we calculated a single PRC and its Sensitivity, using method described above. Because our simulations indicated that the most accurate phase resetting curves are the ones obtained using the lowest noise amplitudes and pulse durations, but our experimental data showed that about 60 pA currents were necessary to get a good PRC with pulse durations of 0.5–1.0 ms, we used the PRC calculated with these stimulus parameters for each cell. We then used the Ermentrout et al. approximation and that particular PRC to derive the predicted relationships between noise amplitude, pulse duration and CV. We measured the goodness of fit to experimentally measured CV values using the proportion of the variance in the data accounted for by the approximation.

An example from a representative STN cell is shown in Figure 5A, for a range of noise amplitudes at a pulse duration of 1 ms. The predicted relationship between CV for this particular cell, based on its spontaneous firing rate and sensitivity measured from its phase resetting curve is shown in red. The approximation in this case produced a very accurate prediction, accounting for about 99% of the variance of CV, even with the largest noise amplitudes at which the cell was firing very irregularly. Similar results were obtained for the other neurons, as indicated by the histogram of R^2^ values for the entire sample shown in Figure 5B. Even for the cells with the poorest fits, the phase-model based approximation accounted for about 80% of the variance in CV for the experimental data.

We took a similar approach to the effect of current pulse duration. The approximation to the phase model predicts a square root relationship between pulse duration and CV. The effect of pulse duration on CV in the same neuron is shown in Figure 5B, showing a range of pulse durations taken at 60 pA. The histogram shown in Figure 5C gives the R^2^ values for the fit of the approximation to the duration data in the entire sample, measured at a σ_Noise_ of 60 pA as in Figure 5B. As for noise amplitude, all cells were well fit by the phase model-based approximation. It is worth repeating that these are not curves fit to the data post hoc, but are predictions of the data points made in advance of their measurement, based on the stimulus parameters, the cell's average firing rate, and its PRC.

**Figure 6:**
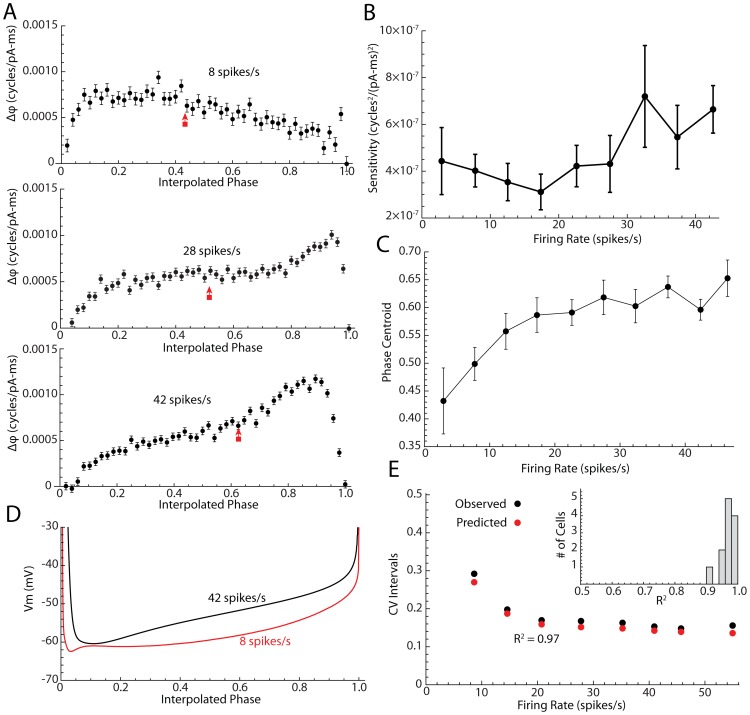
Prediction of CV in STN neurons with changes in firing rate. A. PRCs calculated for a single STN neuron at three different rates. In all neurons, large increases in firing rate by injection of constant current resulted in a shift of the peak of the PRC to later phases, and an increase in the overall amplitude of the PRC. Centroids of the PRCs are indicated by red arrows. B. The changes in mean Sensitivity (integral of the square of the PRC) with changes in current imposed by constant current injection for the sample of 12 neurons. C. Average phase centroid versus mean rate in the same sample. D. Changes in average interspike interval membrane potential trajectory with rate in the same cell shown in A. E. Fit of the phase neuron approximation for CV for the same neuron, based on the experimental value of the PRC sensitivity for each firing rate. Inset is the histogram of R^2^ for the fit obtained for each cell in the sample.

In a separate sample of 12 STN neurons, we measured the response to noise at a range of firing rates, which we adjusted by passing constant current through the recording electrode. Constant current was varied from −50 pA (which was near the point at which firing failed) to +200 pA (which produced sustained firing at about 50 spikes/s). As previously reported [17], STN neurons undergo a slow but powerful spike frequency adaptation when driven to fire at high rates. In all cases, we delayed collecting our responses to noise injection until the cells' firing rates had stabilized after each change in constant current (which took approximately 30 seconds). The results of these experiments are shown in Figure 6.

Although small changes in firing rate did not produce alterations in the PRCs, larger rate changes altered the PRC dramatically, as shown in Figure 6A. There were two kinds of changes seen at high firing rates. The shape of the phase resetting curve changed, and the change was always a shift in the peak of the curve toward later phases (analyzed in more detail below). The second change was an increase in the overall size of the PRC, reflected in an increase in Sensitivity, as shown in Figure 6B (F = 2.81, df = (7,77), p<0.05). Over the normal range of spontaneous firing rates for STN neurons (8–30 spikes/s), the overall size of the PRC was insensitive to changes in firing rate. This is in agreement with our observation that PRC shape and Sensitivity were not correlated with the cell-to-cell variation in spontaneous firing rate (see above). There was sometimes (but not always) an increase in Sensitivity at very low rates as well, but when present this occurred at rates near those at which repetitive firing failed, and was replaced by rhythmic bursting [17].

There was also a systematic change in the shape of the PRC with firing rate increases produced by constant current injection. Increasing rate shifted the peak of the PRC to the right, as shown in Figure 6A. We calculated the phase centroid (the first moment) of the PRC by summing the product of the PRC values and their phases and dividing by the sum of PRC values. Analysis of variance for the effect of injected current on the phase centroid in Figure 6C confirmed that the systematic shift in the shape of the PRC was statistically significant (F = 13.39, df = (7,77), p<0.01). The change in the shape of the phase resetting curve was also reflected in the interspike trajectory of membrane potential, as shown in Figure 6D. At higher firing rates, the membrane potential trajectory shifted from the concave-up shape seen in most STN neurons during autonomous activity, to a more ramp-like, or concave-down shape. The slightly concave-down phase trajectory in the example in Figure 6D (black trace), has its shallowest slope late in phase (between 0.7 and 0.95) corresponding to the peak in its phase resetting curve. In contrast, the shallowest slope in the concave-up trajectory at the low rate (red trace in Figure 6D) occurs at earlier phases (between 0.2 and 0.5), and the corresponding phase resetting curve peaks earlier. The gradual fall of the phase resetting curve at phases between 0.5 and 1 corresponds to the gradual increase in slope of the membrane potential trajectory, whereas at higher rates the slope of the trajectory suddenly increases just before the action potential, and the phase resetting curve shows a sudden plunge to zero. In addition to these changes in shape, there was a positive shift in the entire trajectory of the membrane potential when the cell was driven to fire at high rates with constant current. Because the threshold for action potentials was shifted less than the rest of the trajectory, this produced a decrease in the overall range of membrane potentials visited during the interspike interval (e.g. Figure 6D).

Because of the changes in Sensitivity depending on firing rate, it was impossible to use a single value to predict the coefficient of variation of firing attributable to the current pulses, as we did for noise amplitude and pulse duration. Instead, we measured the PRC for each firing rate, calculated the Sensitivity for that firing rate, and used that in the Ermentrout et al. approximation to predict the CV. This calculation for the same example shown in Fig. 6A&D is shown in Figure 6E. We calculated the proportion of the variance in CV of interspike intervals accounted for by the prediction done this way in each of the 12 cells. The distribution of those is shown in the inset in Figure 6E. The approximation accounted for at least 90% of the variance in all cells of the sample.

These results indicate that the irregularity of firing of STN neurons subjected to injected noise can be predicted from a simple approximation derived from an idealized phase resetting model of the neuron. A single PRC was sufficient to represent the neuron over a wide range of noise amplitudes and pulse durations, but changing firing rate using constant current altered the PRC, so the prediction required a family of PRCs representing different firing rates.

We have previously shown that firing rate increases in the range outside the normal spontaneous firing range of STN neurons activate a slowly-developing potassium current that is responsible for spike frequency adaptation in these neurons [17]. In our experiments, this current and the spike frequency adaptation was present and at steady state at the time we measured both the PRC and the CV of intervals. We considered the possibility that the changes in PRC size and/or shape we observed might be caused by the conductance change associated with that potassium current.

### Effect of Rate-Neutral Somatic Conductance on the PRC and Membrane Potential Trajectory

**Figure 7:**
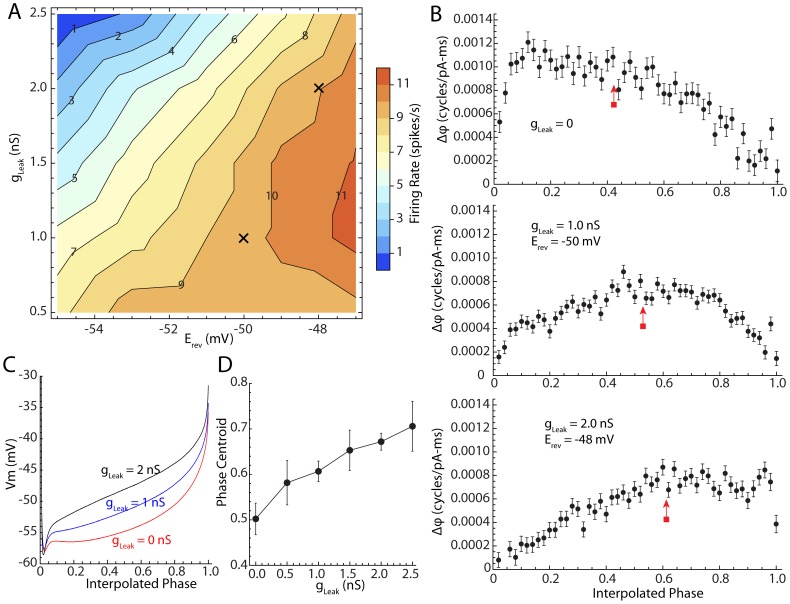
The effect of artificial rate-neutral conductance increases. A. Contour diagram of firing rate varying conductance clamp-applied somatic conductance at a range of subthreshold reversal potentials. X marks two rate-neutral points at 1 and 2 nS conductance levels. B. PRCs calculated at the with rate-neutral applied conductances marked in A. Centroids of the PRCs are at the red arrows C. Membrane potential trajectories corresponding to the PRCs shown in B. D. Average (and standard errors) of the PRC centroid for 9 neurons tested with varying but rate-neutral values of g_Leak_.

To separate the effects of background conductance and firing rate, we applied a background conductance using dynamic clamp, adjusting its reversal potential so that it produced no change in firing rate. Steady state firing at 50 Hz produces a K^+^ adaptation current of about 100 pA at −60 mV, which corresponds to a conductance of about 2.5 nS [17]. We started by scanning a range of artificially applied leak conductances and reversal potentials in this range to determine their effects on firing rate. We used reversal potentials corresponding to the subthreshold range of the interspike membrane potential trajectory. This gave us a map of firing rate over a range of reversal potential-conductance pairs. An example showing the results of this for one cell is shown in Figure 7A. That figure shows a contour map of firing rate for various values of applied (leak) conductance and reversal potential.

As leak conductance was increased at the most hyperpolarized reversal potentials, spontaneous firing slowed, and with sufficiently strong applied conductance, activity ceased. At more depolarized reversal potentials, firing rate increased with increasing leak conductance. For conductances in the range used here (0.5–2.5 nS), there was always a range of subthreshold reversal potentials that allowed autonomous firing to continue at nearly its baseline rate (in this case between 9 and 10 spikes/s). Two example combinations of leak conductance and reversal potential at which firing was maintained a control rates are each marked with an x In Figure 7A. Phase resetting curves collected in the presence of these constant conductances are shown in Figure 7B. Average membrane potential trajectories for these values, and one for control firing in the absence of applied conductance are shown in Figure 7C. Unlike the trajectories observed during applied depolarizing current, applied conductance had little effect on the membrane potential attained during the fast afterhyperpolarization immediately after the action potential. Artificial leak conductance also had no effect on the firing threshold, so the membrane potential range traversed between action potentials was not altered. However, the shape of the membrane potential trajectory was changed in a way similar to that seen during applied constant current. Addition of a rate-neutral leak conductance shifted the membrane potential from a deep scoop shape toward a ramp-like shape, and could even produce a concave-down trajectory as seen during high frequency firing to constant current. We applied pulsed current noise over a range of these frequency-neutral conductance-reversal potential pairs to determine the effect of applied conductance on the PRC. Addition of a leak conductance shifted the shape of the PRC from one that peaked early and gradually decayed to zero to a more symmetric shape, and ultimately to one that peaked at late phases, as shown in the example in Figure 7B. These experiments were performed in 9 neurons, and although different neurons had differently-shaped PRCs in the absence of artificial conductance increases, they all systematically shifted their peak to later phases as conductance was added. An analysis of variance for the 9 cells at values of 0, 1, 2, and 3 nS showed that the right shift in the centroid of the PRC was statistically significant for the sample (F = 80.6, df = 3,27, p<0.01). Unlike the response to constant current injection, there was no increase in the overall amplitude of the PRC as applied leak conductance was increased. Thus the change in PRC shape seen at increased firing rates in STN neurons may be attributed to the increased whole cell conductance generated by the K^+^ spike-frequency adaptation channel. In contrast, the increased sensitivity of the cells, as indicated by the integral of the squared PRC, or the net depolarization of the membrane potential trajectory seed during current injection cannot be attributable to the conductance of the adaptation channel.

## Discussion

Studies of temporal synaptic integration have historically focused on preparations in which neurons have stable resting potentials, and must be driven to fire either by individual large excitatory synaptic events, or by the temporal confluence of many small ones. For firing patterns generated by synaptic excitation from a stable rest state, the influence of the cell's intrinsic dynamics is relatively simple and confined to the time scale of the membrane time constant, with some additional influence from the recent history of spiking (e.g. [18], [19]). In contrast, studies of temporal integration in repetitively firing neurons have focused on the importance of intrinsic neuronal dynamics (see review by Smeal et al. [7]), and often yield complex and intuition-defying results (e.g. [20], [21]). Phase resetting methods show promise as a way to condense the complexity of intrinsic cellular dynamics in repetitively firing neurons, but their experimental use is impeded by the fact that phase is not directly measurable in neurophysiological studies. The need to use time as a surrogate for phase has restricted phase resetting methods mostly to either brief and isolated stimuli, or to temporally extended stimuli that are (or can be treated as) asymptotically small (e.g. [2], [22], [23]). Under these conditions, the cells' firing remains highly rhythmic, and that rhythmicity maintains the relationship between phase and time. This has given the impression that phase resetting techniques are only applicable to rhythmically firing neurons. If this were true, it would be a serious limitation. Encoding information in a neuron's activity depends on the disruption of rhythmic firing; the potential information content of a spike train is inversely related to its periodicity. The experiments reported here were designed to test the usefulness of the phase-resetting approach to predict the firing of oscillatory neurons that are so densely perturbed that they are no longer firing rhythmically. We used symmetric noise to disrupt the relationship between time and phase on individual interspike intervals while maintaining that relationship on average across the entire spike train. We took advantage of a previously published result predicting the variability of a densely perturbed repetitively firing neuron during application of symmetric current noise [12].

### The STN Neuron and Its PRC

The STN is a component of the basal ganglia circuit. It is a compact nucleus consisting entirely of projection neurons, whose axons project to other components of the basal ganglia, most notably the globus pallidus and substantia nigra [24], [25], [26]. The neurons of STN have few or no local axon collaterals [25], and so are apparently not synaptically coupled. They are autonomous oscillators that fire continuously, even when completely disconnected from their synaptic inputs (for review see [14]). Their firing under these conditions is driven by a persistent sodium current that is active throughout the interspike interval membrane potential trajectory, and by action potential afterhyperpolarization currents that include fast and medium components [27], [28]. STN neurons receive excitatory afferents from the thalamus and cerebral cortex and a dense inhibitory input from the external segment of the globus pallidus. Every STN neuron receives approximately 900 synaptic inputs from the globus pallidus alone, arising from a large number (nearly the same number) of different afferent neurons [29]. Most of these normally fire tonically at high rates (up to 100 spikes/s for the pallidal inputs), and because of synaptic depression at those rates are individually very weak [30]. STN neurons are tonically active in vivo, firing about the same rate as they do when isolated, although their firing in vivo is much more irregular, presumably because of the ongoing synaptic bombardment [14]. This makes the STN neuron a good subject for the study of spike timing in densely perturbed oscillatory cells, but whether they normally encode their inputs by variations of timing during repetitive firing, or by the insertion or deletion of spikes in the spike train is not known. The mechanism of repetitive firing in the STN neuron makes it especially suited for our analysis. The afterhyperpolarization currents are brief compared to the interspike interval of the neuron when firing at its spontaneous rate. The persistent sodium current controlling its autonomous oscillation activates and deactivates rapidly, and the cell has no prominent subthreshold resonance. Together these ensure that the cell's membrane potential changes more slowly than changes in ion channel activation over most of the interspike interval. As a result, membrane potential trajectory and PRC are predictable from the steady-state I–V relationship over that portion of the interspike interval not dominated by afterhyperpolarization currents [11].

### Heterogeneity of Subthalamic Neurons

STN neurons are usually considered to belong to a single physiological cell type, despite the existence of subtypes based on axonal branching [26], [25] and variations in physiological properties [27], [31], [32]. Our results suggest that the shape of the PRC may vary substantially among STN neurons. The variation in shape we observed was not arbitrary. For example, all subthalamic PRCs we measured were of type 1, i.e. they were non-negative everywhere. However, they showed substantial differences in the phase of their maximal sensitivity, and in overall sensitivity to injected current. These variations were similar to those seen for individual neurons made to fire outside their normal range of rates, but in this case were not attributable to differences in firing rate. Even so, the differences in PRC shape reflect difference in interspike interval membrane potential trajectory, and so (like the rate-induced changes) probably reflect differences in the relative contributions of the several ionic conductances that contribute to the STN cell's autonomous activity. Ion channels in STN neurons, including those responsible for spontaneous firing, are subject to modification by neuromodulators [14]. If there is a similar variation in vivo, it could produce substantial heterogeneity in the physiological responses of STN neurons to their inputs, and contribute to the decorrelation of activity in the STN-GPe loop [33]. The shape of the PRC also determines the phase at which cells tend to fire relative to entraining periodic components in the overall synaptic input current (e.g. [34]), and so this kind of heterogeneity among cells may be important in generating population activity patterns of STN cells in response to rhythmic components of their cortical and pallidal afferents.

### The PRC and Membrane Potential Trajectory

Like motoneurons [35], [36] and many other neurons, the interspike membrane potential trajectory of STN neurons may consist of two portions, and initial scoop-shaped part following the action potential, and a later ramp-shaped portion that leads up to the sudden rapid depolarization to the firing point. The hyperpolarizing onset of the scoop gets its shape from the spike afterhyperpolarization currents. As the afterhyperpolarization currents subside, the membrane potential turns in the depolarizing direction. For STN neurons, the trajectory taken by the membrane potential for times after the afterhyperpolarization is complete but before the cell fires again is determined by the shape of the steady-state I–V curve. For cells like this, whose firing arises from a saddle node bifurcation, the I–V curve must be negative everywhere in the subthreshold range to sustain repetitive firing. The slope of the I–V curve determines the curvature of the membrane potential trajectory. Where the I–V curve is nearly flat, the membrane potential has constant slope, and resembles a ramp. Where the I–V curve has a positive slope, the trajectory of the membrane potential is concave-down, and where the slope conductance is negative the membrane potential trajectory is concave-up. For most subthalamic neurons firing autonomously, the membrane potential follows a concave-up scoop-like trajectory between action potentials (e.g slow firing in Figure 6C). The PRC is approximately proportional to the reciprocal of the slope of the membrane potential trajectory [11]. Thus for most subthalamic neurons firing at their spontaneous rates, the PRC reaches its maximum early in the interspike interval, and gradually approaches zero.

Small changes in rate had little effect on the shape of the membrane potential trajectory or the PRC of STN neurons. Large increases in firing rate resulted in changes in membrane potential trajectory similar to those seen in the secondary and tertiary range of firing in motoneurons [35]. As rate increased, the later part of the membrane potential trajectory gradually transformed into a linear ramp shape, with a more sudden depolarization preceding the action potential. The peak of the PRC likewise shifted to later phases and its decrease at late phases became more sudden. At high rates the membrane potential trajectory became mostly concave-down, and the peak of the PRC moved toward the late phases, with a sudden crash to zero at the end.

These changes in membrane potential trajectory and PRC could be duplicated by increasing the membrane conductance in a rate-neutral fashion, suggesting that they are caused by accumulation of afterhyperpolarization currents that change the shape of the steady state I–V curve. The fast and medium afterhyperpolarization currents in STN neurons do not accumulate [28], but there is a very slowly developing potassium conductance responsible for their powerful slow spike frequency adaptation [17]. Adding rate-neutral conductance adjusted to match that responsible for spike frequency adaptation reproduced the entire sequence of changes in membrane potential trajectory and PRC shape with applied conductance alone. As expected from the study of the steady state I–V curve of the STN neuron [37], [38], addition of a powerful positive-slope conductance competes with the Na^+^ conductance responsible for the negative slope of the I–V curve at more hyperpolarized potentials early in the trajectory more than at more depolarized potentials late in the trajectory, when the Na^+^ current is stronger. This moves the subthreshold maximum of the I–V curve (and inflection point of its slope) to the right, making early parts of the trajectory concave-down and shifting the peak of the PRC to the right. With sufficiently high conductance, so that the negative slope conductance occurs just at the action potential onset, the membrane potential trajectory is gradually transformed in the direction of the concave-down form expected for a repetitively-firing leaky integrate and fire neuron. The PRC of the cell subjected to a powerful leak conductance (at very high rates) likewise resembles that of the leaky integrate-and-fire neuron, which is low everywhere except at the latest phases, where it rises exponentially and falls with a discontinuity at phase = 1.

The application of a background conductance, as used here, also approximates the state of balanced excitation and inhibition that might occur in vivo. Neurons in vivo fire at mean rates similar to those in slices, despite the larger volume of synaptic barrage. Perhaps synaptic excitation and inhibition are approximately balanced in vivo, leading to a net conductance with reversal potential that allows continuous repetitive firing, as observed in our conductance clamp experiments. Rapid fluctuations in synaptic input could produce current noise similar to the noise we applied, to create the irregular firing pattern normally observed in vivo. The shape of the PRC and the cell's overall sensitivity would be determined by the average conductance generated by the balance of synaptic inputs, while the moment-to-moment variation of spike times would arise from its interaction with the fine structure of synaptic input changes.

### Sensitivity Changes and Afterhyperpolarization Currents

In addition to the changes in shape of the PRC as rates were increased, STN cells showed an increase in overall sensitivity to noise when firing at high rates. This change, an increase in the overall amplitude of the PRC, was not mimicked by increasing the conductance of the neuron, and was so not attributable to the slow spike frequency adaptation conductance seen in STN cells. However, it was reflected in the membrane potential trajectory, in the form of a general depolarization of the membrane potential throughout the interspike interval. A potential mechanism for this change is the depression of the fast afterhyperpolarization, and saturation of the medium afterhyperpolarization previously observed for STN neurons [28]. The reduction in spike-locked afterhyperpolarization currents may reduce the strength of the membrane potential reset following each action potential, and so also reduce the potential range that must be traversed from the end of each action potential to the threshold for the next. Like the buildup of the slow adaptation conductance, these changes in ion channel properties alter the current balance equation and the limit cycle of the neuron in a rate-dependent manner. The PRC changes accordingly. Our method of measuring the PRC from injected noise allowed its re-evaluation as these changes occurred, and when they were taken into account, the predictions of the phase model remained accurate.

### The Phase Model

The advantage of the phase model is that it is simple but retains neurophysiological validity. The phase model is a simplification derived from a well-established theoretical understanding of neuronal dynamics (see review by Smeal et al. [7]). The PRC is experimentally measurable with no other free variables to be estimated separately or inferred post hoc. The principal disadvantage of the phase model is that it is not a complete description of the neuron, but rather a description of the neuron's state space in the vicinity of a single closed trajectory. If the neuron's average trajectory changes, for example because of a change in the ionic conductances participating in repetitive firing, its PRC may change as well (e.g. 5). A single PRC is not a general model for describing the full range of potential firing patterns or stimulus encodings a neuron may express; a family of such PRCs is required. For these reasons, the phase model is most easily applied to the study of neurons responding to their inputs with short-term changes in the timing of action potentials while maintaining a constant average firing rate.

### Irregular Firing Does Not Mean the Cell Is Not Oscillating

Our findings confirm previous theoretical studies [e.g. 39] showing that the phase model predicts spike timing of repetitive firing neurons in response to complex input patterns even when the cells are so densely perturbed that no rhythmicity can be detected in their firing (e.g. using the autocorrelation histogram). If one observed this firing pattern in vivo, not knowing anything about its origin, it would be impossible to recognize it as a densely perturbed oscillation. However, there can be no doubt that the cell continued to oscillate deterministically in these perturbed conditions, because we could predict the precise timing of action potentials from knowledge of the unperturbed oscillation, and we could recover the PRC from the perturbed firing pattern by multiple regression with the exact pattern of injected noise. The recovered PRC in this case was effectively the same one obtained at lower levels of noise, or even using single synaptic stimuli occurring only once in an interspike interval. The apparently random firing pattern obtained when the cell was densely perturbed was accurately predicted from the deterministic interaction of the injected pattern of current pulses and the mechanism of autonomous oscillation of the neuron as it is in the absence of input.

The accuracy of the phase model in densely perturbed cells was remarkable, with the first order PRC accounting routinely for 80% of the variance in the timing of action potentials. The accuracy of the phase model's prediction of changes in interspike interval variance with noise amplitude is even more remarkable considering that it was not based on the full model, but rather on an approximation that is only expected to be accurate at low noise levels. Our results suggest that the phase model and the Ermentrout et al. approximation are robust to violation of their assumptions under real life conditions, at least in STN neurons.

## Methods

### Ethics Statement

All experiments were conducted in accordance with the NIH guidelines and were approved by the Institutional Animal Care and Use Committee of the University of Texas at San Antonio.

### Slice Experiments

Experiments were performed on 89 cells recorded from slices cut from the brains of Sprague-Dawley rats aged 16–35 days. All experiments were conducted in accordance with the NIH guidelines and were approved by the Institutional Animal Care and Use Committee of the University of Texas at San Antonio. Rats were deeply anesthetized with isoflurane and perfused intracardially with sodium-free ice-cold artificial cerebrospinal fluid consisting of (in mM) 2.5 KCl, 1.25 NaH_2_PO_4_, 0.5 CaCl_2_, 10 MgSO_4_, 10 D-glucose, 26 NaHCO_3_, and 202 sucrose, at pH 7.4. The brains were removed and sectioned in the parasagittal plane at 300 µm, and slices containing the subthalamic nucleus were collected in artificial cerebrospinal fluid containing containing (in mM) 126 NaCl, 2.5 KCl, 125 NaH_2_PO_4_, 2 CaCl, 2 MgSO_4_, 10 D-glucose, and 26 NaHCO_3_, 0.005 L-glutathione, 1 Na-pyruvate, and 1 Na-ascorbate, and bubbled with 95% O_2_-5% CO_2_. Slices were warmed to 35°C for one hour after cutting and stored at room temperature until used. For recording, slices were superfused on the microscope stage with artificial cerebrospinal fluid bubbled with 95% O_2_-5% CO_2_ at 34°C in the microscope chamber.

All recordings were performed in the perforated patch configuration. This was required to maintain consistent rhythmic autonomous firing in STN neurons. In whole-cell recordings, firing gradually slowed, became irregular, and ultimately failed. Our experiments required firing at a consistent rate for greater than 30 minutes. In perforated-patch recordings, cells maintained their baseline firing with little or no change in rate or regularity for more than an hour. Perforated patch recordings were made with glass micropipettes pulled on a Sutter P-97 puller and filled with a solution containing 140 mM Na-methylsulfate, 10 mM HEPES, 7.5 mM NaCl. One ml of this solution was filtered and then and 1 µl of 0.5 mg/ml gramicidin (invitrogen) in DMSO added so that the final concentration of gramicidin was 0.5 µg/ml and DMSO 0.1%. This solution was mixed thoroughly but not filtered. In most experiments, 20 µM Alexa Fluor 594 biocytin was added to the electrodes. Absence of fluorescence in the recorded cell was used to verify that the patch had not ruptured and the cell had not been dialyzed by the contents of the electrode. Electrodes had resistances of 3–7 Megohms. After establishing a seal, 10–30 minutes were allowed for the establishment of sufficient access for current clamp recording (20–70 Megohms). Recordings were made using an Axon Instruments Multiclamp 700B amplifier in current clamp configuration, with the output filtered at 10 kHz. Data were acquired using a HEKA Instruments (New York NY) ITC-18 A/D converter sampling each channel at 20 kHz. Current waveforms for intracellular injection were generated and applied, and recordings made using custom software written using Igor Pro (WaveMetrics, Portland OR). A second computer running RTXI (the Real-Time eXperiment Interface; www.rtxi.org) and using a National Instruments (Austin TX) PCIe-6251 A/D board was also present and digitizing the same waveforms. For conductance clamp experiments, this system was programmed to apply a variable leak conductance, and the current output from that system was summed with that from the ITC-18 using a software mixer implemented in RTXI.

Because our goal was to quantify the relationship between applied current and spike timing, special care was taken to ensure that the capacitance of the electrode and stray capacitance of the amplifier headstage were optimally compensated. We used current pulses, rather than bandpass limited white noise, because we could use the membrane potential transients to continuously monitor the capacitance compensation. If the capacitative transient could not be limited to a single sample (50 µs), using the compensation controls, we discarded the electrode. We also monitored the responses to current pulses to ensure that the membrane potential response was primarily capacitative (i.e. the transients were linear ramps lasting the duration of each pulse). This limited the maximum duration of current pulses to about 5 ms. Data used in this report all employed pulses durations of 2 ms or less. Episodes of data had durations of 60–120 seconds, and during this time current pulse duration was fixed, but each pulse was an independent draw from a Gaussian distribution with mean zero. For each episode, the standard deviation of the pulse amplitude distribution was fixed. On successive episodes, the pulse duration or standard deviation of the pulse amplitude distribution was altered. In some experiments, constant current was passed to alter the firing rate of the neuron. In these cases, data were not collected until spike frequency adaptation was complete and the cell had assumed a constant firing rate. Usually, it took approximately 30 seconds to achieve a steady firing rate after a change in constant current. Firing rate and coefficient of variation were monitored in real time using a module created for this purpose using RTXI. Data analysis was performed using routines written in Mathematica (Wolfram Research, Champaign IL).

### Phase Model Simulations

Phase model simulations used a simple first order method implemented as a custom program written in C. Trajectories started at phase zero and for each time step of width Δt, the value of the phase φ_t_ was updated according to
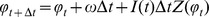
in which1/ω is the average period and Z(φ) is the infinitesimal PRC. The time of the next spike is determined as the time when the phase φ_t_ equals 1.

The PRC used for Monte Carlo simulations was obtained from a minor variant of the Traub model, as described previously [13]. For convenience, the average period was set to be 1 sec and the time step was 1 ms. Current pulse amplitudes were drawn from a Gaussian distribution generated using the Box-Muller method [40]. When the phase of each trajectory crossed one, the time of firing was recorded, application of noise was discontinued, but the phase continued to advance under the influence of drift until all trajectories in the simulation had fired. This allowed us to creating phase distributions at all relevant time points, used, for example, as in Figure 1A. Each simulation consisted of 5000 trajectories.

### The Noise Regression Method for Measuring the PRC

We modified a method outlined by Netoff et al. [16] for constructing an infinitesimal PRC from the response to injected Gaussian noise. Each interspike interval, indexed by α, is divided into a number of equally spaced phase bins, indexed by i. We then integrate the noise current injected during that bin on that trial to obtain an amount of charge delivered, Q_α,I_. The number of phase bins used was equal to the mean interspike interval divided by the pulse size (to get on average one noise pulse per bin), or 50, whichever was smaller. The goal was to have phase bins at least as large as the duration of a noise pulse, to maintain statistical independence between the current in each bin.

The vector of charge delivered across bins is viewed as the independent variable in a multiple linear regression, with the duration of the interspike interval (normalized by the average interval) serving as the dependent variable. The model for the regression is:
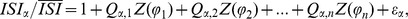
where ε_α_ represents residual error and Z(φ_i_) are the regression coefficients to be derived. These coefficients represent the values of the PRC at the center of each phase bin and are expressed in units of Δphase per unit charge delivered (the correct units for the infinitesimal PRC). Using standard linear regression (Mathematica, Wolfram Research, Champaign, IL) the PRC is calculated as
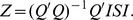
Standard errors for the estimates of Z are calculated as the diagonals of the matrix (Q′Q)^−1^.This method was outlined by Netoff et al. [16], but they did not present any application of the method to experimental data.

**Figure 8:**
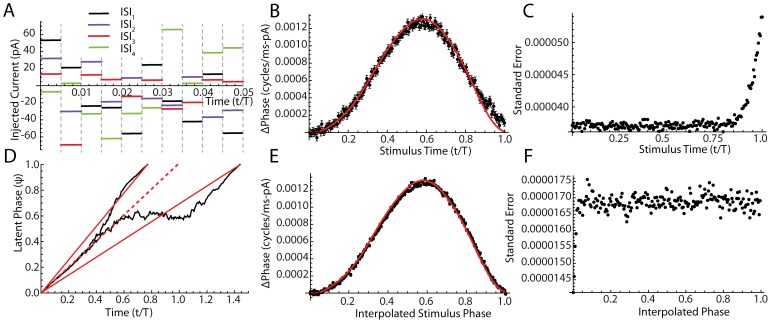
The multiple regression method for calculating the PRC, and the choice of phase interpolation. A. The use of pulsed noise provides a natural set of independent stimuli at each time slice (corresponding to pulse duration) during the ISI. Different ISIs are indicated by different colors. B. The PRC and its standard errors calculated by multiple regression on a Monte Carlo simulation using fixed time steps and phase interpolation based on the mean ISI. The true PRC used by the simulation is shown as a red line. Note the consistent error near the end of the PRC. C. The standard error of the PRC estimates as a function of phase. The error increases dramatically at large phases. D. The strategy for phase interpolation. Two phase versus time trajectories from the Monte Carlo simulation used in B and C are shown in black. One is the trajectory a very short ISI, and one for one of the longest ISIs in the simulation. The linear phase versus time estimate used for calculating the phase of current pulses based on mean interval is shown as a dotted red line. This estimate is good at early times in the ISI but fails at longer times. The phase interpolations we employed is shown as solid red lines. Although less accurate at short times they are more accurate later, at times when the PRC values are higher and the noise is more influential. E. The PRC calculated using our interpolation method, overlaid by the true PRC for the simulation (red). F. The standard error of the estimates of the PRC in E. Note lower overall error, and the even distribution of error across the ISI.

As a proof of concept, we used Monte Carlo simulations of a phase model to test whether this noise-based regression method could accurately estimate a known PRC, shown in Figure 8A–C. We used the same PRC as in figure 1, and injected repeated patterns of pulsed noise as in Figure 8A. The traditional method for calculating the PRC uses the time since the previous spike as an estimate of phase. So in our regression method, the ith phase bin reaches from T(i-1)/n to Ti/n, where T is the average period of the neuron (equal to the mean of the ISI distribution). Figure 8B shows estimated PRC along with estimated errors with the actual PRC superimposed in red. The standard errors for the estimated PRC are shown in figure 8C.

All methods of experimentally estimating the phase resetting curve are troubled by the decoupling of phase and time that occurs within each interspike interval. Because there is always some background noise in real neurons, this problem attends all methods of calculating the phase resetting curve, even those in which a single current pulse is injected on each ISI. Methods using the time since the previous spike as an estimate of latent phase are challenged by the accumulation of variance in the phase distribution late in the interspike interval (see Figure 1B) [41], . This results in the sharp increase in PRC error near the end of the ISI in figure 8C.

To partially account for the drift in phase during each ISI, we modified the method to map time to phase based on the length of each interspike interval, rather than based on the average period of the oscillation. We define the Interpolated Phase at any time between two spikes as the time since the previous spike divided by the length of that particular interspike interval. The Interpolated Phase indicates the proportion of the interval that has elapsed up to that time, shown in Figure 8D. The error of phase estimation is not completely removed, but is distributed more equally over the ISI and is reduced overall. Using Interpolated Phase instead of the time since the previous spike, we define a phase bin as lasting from time T(i-1)/n to Ti/n, where T now set equal to the length of each individual ISI, instead of the average period of the neuron. We then calculated the charge delivered in each phase bin, and performed a multiple linear regression as outlined above. As shown by comparing panels B&C with E&F in the example in Figure 8, the use of interpolated improves the estimate of the PRC by more than a factor of 2 throughout the interval, and does not suffer from a sharp increase in error near the time of the next spike. The Interpolated Phase approximation works well in our setting because alterations in phase due to perturbations at any given time are small, and positive and negative perturbations roughly cancel throughout the interval so that Interpolated Phase is a reasonable first order approximation to the actual latent phase for any given interval.

In these Monte Carlo simulations, the only source of noise was the injected current and the Interpolated Phase approximation works best at very low noise levels. For experimentally measured PRCs, there was a limit on how small noise levels could be. This limit was set by the intrinsic variability of ISIs, which is independent of the injected current. In this case, the proportion of the variance in ISIs that could be attributed to the injected noise interacting with the PRC was obtained from the R^2^ for the multiple regression. We calculated the PRC from episodes collected using the smallest value of noise consistent with a good estimate of the PRC, as indicated by the R^2^ for the regression.

### Using the PRC to Predict Interspike Intervals

Having obtained a PRC in the above manner, we could then predict interspike intervals from other segments of data collected from the same cell. We used linear interpolation between bin centers to obtain a function Z(φ) that is a continuous function of phase. We assumed Z(0) = Z(1) = 0 to continue the interpolation past the first and last bin centers. We then used the dynamic phase model with a time step Δt = .05 ms and injected current matched to the experimentally delivered sequence of current pulses to derive a predicted phase trajectory. The time when this trajectory crossed 1 served as the predicted time of the next spike.
